# Central Nervous System Involvement as Outcome Measure for Clinical Trials Efficacy in Myotonic Dystrophy Type 1

**DOI:** 10.3389/fneur.2020.00624

**Published:** 2020-10-07

**Authors:** Costanza Simoncini, Giulia Spadoni, Elisa Lai, Lorenza Santoni, Corrado Angelini, Giulia Ricci, Gabriele Siciliano

**Affiliations:** ^1^Department of Clinical and Experimental Medicine, University of Pisa, Pisa, Italy; ^2^IRCCS S.Camillo Hospital, Venice, Italy

**Keywords:** myotonic dystrophy type 1, central nervous system, clinical trials, neuropsychological outcome measures, neuroimaging

## Abstract

Increasing evidences indicate that in Myotonic Dystrophy type 1 (DM1 or Steinert disease), an autosomal dominant multisystem disorder caused by a (CTG)n expansion in DMPK gene on chromosome 19q13. 3, is the most common form of inherited muscular dystrophy in adult patients with a global prevalence of 1/8000, and involvement of the central nervous system can be included within the core clinical manifestations of the disease. Variable in its severity and progression rate over time, likely due to the underlying causative molecular mechanisms; this component of the clinical picture presents with high heterogeneity involving cognitive and behavioral alterations, but also sensory-motor neural integration, and in any case, significantly contributing to the disease burden projected to either specific functional neuropsychological domains or quality of life as a whole. Principle manifestations include alterations of the frontal lobe function, which is more prominent in patients with an early onset, such as in congenital and childhood onset forms, here associated with severe intellectual disabilities, speech and language delay and reduced IQ-values, while in adult onset DM1 cognitive and neuropsychological findings are usually not so severe. Different methods to assess central nervous system involvement in DM1 have then recently been developed, these ranging from more classical psychometric and cognitive functional instruments to sophisticated psycophysic, neurophysiologic and especially computerized neuroimaging techniques, in order to better characterize this disease component, at the same time underlining the opportunity to consider it a suitable marker on which measuring putative effectiveness of therapeutic interventions. This is the reason why, as outlined in the conclusive section of this review, the Authors are lead to wonder, perhaps in a provocative and even paradoxical way to arise the question, whether or not the myologist, by now the popular figure in charge to care of a patient with the DM1, needs to remain himself a neurologist to better appreciate, evaluate and speculate on this important aspect of Steinert disease.

## Introduction: The Complexity of a Disease

Myotonic dystrophy type 1 (Steinert disease) is an inherited, slowly progressive, autosomal dominant disorder, representing one of the most common neuromuscular diseases with a frequency variable in different geographical areas, of 1–20 per 100,000 inhabitants (1, 2).

The genetic defect consists in an abnormal dynamic repetition of the unstable trinucleotide triplet CTG (cytosine-thymine-guanine) at the level of the 3 'untranslated region of the *DMPK* gene located on the long arm of chromosome 19, in locus 13.3 (3, 4).

The *DMPK* gene encodes for the DMPK (Myotonic Dystrophy Protein Kinase), a serine-threonine kinase with analogies to adenosine monophosphate (cAMP) dependent protein kinases undergoing self-phosphorylation, unlike other protein kinases, this is unable to phosphorylate other membrane proteins. DMPK haploinsufficiency ensues in increased myosin phosphatase phosphorylation, this inhibits its control on phosphorylated myosin levels, the latter which is responsible for increased calcium sensitivity of smooth muscle cells and alterations in the cytoskeleton of non-muscle cells (5, 6).

However, a more general RNA-mediated disease model underlies, as for other hereditary disorders caused by noncoding microsatellite expansions, DM1 molecular pathogenesis. Myotonic dystrophy type 1 is in fact a multisystem disease with a wide intra and inter-individual variability. The symptoms of DM1 can occur at any time of life, from child to adulthood. Patients can have a completely asymptomatic course or present severe phenotype. The wide intra and inter-individual variability observed in this disease is mainly related to the molecular mechanism underlying the pathology, i.e., to the mutational instability of the CTG expansion, which increases in subsequent generations (anticipation phenomenon, which is directly related to the severity of the symptoms and inversely related to the age of onset of the disease) and in the somatic tissues (with different expansion in different tissues and between the various patients) (7).

According to such a model, the numerous and different clinical manifestations in DM1 are determined by a reversion, in adult tissues, to fetal RNA processing patterns due to the increased expression of toxic CUG RNA expansions (8). The etiopathogenetic hypothesis most accredited to explain at the same time multisystem and high heterogeneity of DM1 is that of toxic RNA (“RNA toxic gain of function”). The genetic defect underlying the disease is the abnormal expansion of the CTG microsatellite triplet that is located at the intronic 3′ non-coding sequence of the *DMPK* gene, this resulting in the formation of expanded untranslated CUG RNA transcripts which accumulate in nuclear foci sequestering muscleblind-like (MBNL) proteins and blocking their splicing activity. Therefore, a loss of function of MBNL in DM1 affected tissues occur and more notably a splicing dysregulation of specific MBNL-targeted transcripts with a shift from adult to fetal splicing events for some tissue-specific disease manifestations such as myotonia and insulin resistance (9–12). On the other hand, protein kinase C activation determines increased levels of hyperphosphorylated CUGBP1/ETR3-like factor 1 (CELF1), in affected tissues this inducing with a gain of function mechanism is a reversion to fetal isoforms of targeted proteins in adult DM1 tissues (13). Some more ancillary molecular pathogenic mechanisms are even hypothesized, for instance, the so-called “myomiRs,” a group of non-muscular miRNA playing a role in muscle maintenance and myogenesis, which is also expressed in the brain. Till date, no specific association with the central nervous system involvement has been reported (14). All of these molecular mechanisms can then account for a number of downstream altered molecular pathways, these in turn are responsible of the multiplicity of clinical manifestations of the disease, the knowledge of which can be useful to increase the therapeutic army in addition to the gold standard genetic therapy. This is the case, for instance, of the reported alterations of the cell redox balance and the attempts to treat the ensuing oxidative stress with antioxidant therapies like cysteine dietary donors (15, 16).

However, it is not fully known how the toxic RNA hypothesis leads to the involvement of the central nervous system (CNS). From the neuropathological point of view, the presence of neuronal loss and neurofibrillary aggregates was found in patients with DM1. These characteristics, also present in other neurodegenerative diseases such as Alzheimer's disease, led to hypothesize an altered splicing of the tau protein in patients with DM1 which could explain the accumulation of neurofibrillary plaques. However, an alteration of neurodevelopment has also been hypothesized, which could account for the cognitive behavioral alterations of congenital forms (11, 17–19). On the other hand, from a therapeutic point of view, similarly to skeletal muscle involvement, multiple molecular pathways can be considered in trying to explain alterations of CNS in DM1, this is important in view of clinical trials that should consider this in their targeted objectives and the modifications of the CNS related aspects.

During the years, various clinical classifications of disease have been proposed and in particular the clinical spectrum has been divided into three forms on the basis of severity (moderate, classical and severe) and in five clinical categories based on the age at onset of symptoms (congenital, infantile, juvenile, adult and late-onset form). However, to date there is still no general consensus on the clinical classification of type 1 myotonic dystrophy (20).

### Multisystem Involvement

In considering the aim of this review it seems important to address the specific topic of CNS involvement and give some brief details on other relevant clinical aspects that can interfere with CNS function in a patient with Steinert disease.

**Muscular impairment:** in DM1 patients, muscular impairment is characterized by asymmetrically muscle weakness and atrophy, especially in distal regions of limbs, myotonia and central and peripheral fatigue (21). Early involvement of the face and anterior neck muscles are common (2, 22). Myotonia is one of the cardinal symptoms and is characterized by delayed relaxation (prolonged contraction) of the skeletal muscles after voluntary contraction. Fatigue, described as extreme tiredness resulting from mental or physical exertion or illness, is common, and early symptoms in DM1 seems not to correlate with the progression of muscle weakness (23). Muscle MRI studies revealed more fat infiltration in muscles of patients with DM1 than in unaffected controls, especially in gastrocnemius medialis, soleus and tibialis anterior (24). Recently Solbakken and colleagues showed higher levels of fat infiltration and reduced muscle size in Trunk muscles of DM1 patients with correlations between MRI pictures and deficit in motor performance and respiratory function (25).

**Respiratory involvement**: Pulmonary complications are the leading cause of death in DM1 patients (26). Patients with DM1 tend to exhibit earlier respiratory insufficiency than patients with other neuromuscular diseases (27, 28). Clinicians must monitor issues such as recurrent pneumonia at baseline and serially, with pulmonary function tests. Being a relatively slow progressive disorder, respiratory involvement frequently presents itself with symptoms such as fatigue, excessive daytime sleepiness, sleep disorders (obstructive or CNS mediated sleep apnea); thereafter, clinical manifestations progress to an ineffective cough, respiratory insufficiency (restrictive ventilatory pattern), and recurrent pulmonary infections (26, 29). Many mechanisms are involved to explain respiratory insufficiency in DM1 besides respiratory muscle weakness and chest mechanics, and among those alterations are neural respiratory drive and abnormal central respiratory control (central hypoventilation) (30). A periodic respiratory assessment is recommended with evaluation of pulmonary function on initial assessment, with a pulmonary service referral to assess the need of receiving support for secretion management, non-invasive or invasive ventilation (31).

**Cardiovascular involvement:** Cardiac manifestations are among the most common manifestation of DM1. It can be present in many different forms ranging from dilative cardiomyopathy leading to terminal heart failure, rhythm disturbances, conduction abnormalities, myocardial abnormalities (cardiac non ischemic fibrosis and fatty infiltration), secondary valve insufficiencies. these cardiac abnormalities are potentially life threatening (32). Palpitations, chest pain, dyspnea, orthopnea, lightheadedness, and syncope are common symptoms requiring cardiac investigation, even if sometimes significant cardiac involvement is asymptomatic. These features are the second life-threatening manifestation of DM1 with up to 30% of death in DM1 due to cardiac causes, of which one third are sudden cardiac death as results of conduction block or ventricular tachyarrhythmia (33, 34).

As other disease manifestation, there is a direct correlation between CTG length and cardiorespiratory involvement: longer CTG expansion is associated with arrhythmias as well as PR and QRS prolongation, with lower values for maximal inspiratory pressure and vital capacity, the genetic test is helpful in risk stratification (35, 36). From the time of DM1 diagnosis, a lifelong cardiac surveillance for arrhythmias and cardiomyopathy, and eventually a prophylactic treatment is a major goal in treating DM1 (37). In any case, cardiac involvement can influence the level of patient autonomy and their performances during everyday life activity. Recently, the importance of periodic cardiologic follow up has been underlined by the clinical Care Recommendations. This paper stresses the need of more frequent cardiac imaging and ambulatory monitoring to detect asymptomatic arrhythmias, especially in patients aged >40 years and with 500–1,000 repeats. Accordingly, a primary prevention pacemaker or ICD in a patient who is at high risk of sudden cardiac death should be considered. Therapy with beta-adrenergic blockers, angiotensin-converting enzyme inhibitors, or angiotensin receptor blockers may be considered in patients with reduced ejection fraction (38).

## Clinical Features of CNS Involvement

Many literature studies have documented a selective involvement of neuropsychological functioning regarding attention, executive functions, visuo-spatial and visuo-constructive abilities (39). This affects both the cognitive and behavioral personality domains in the patient. In addition to more traditional neuropsychological functioning, in recent years, research has turned its interest into the study of neuropsychiatric comorbidities and pathological behavioral patterns in DM1, identifying the presence of apathy, lack of interest, decrease of emotional interest in activities and irritability (40–42).

The central nervous system alterations described as above are associated with a range of neuropsychological and cognitive dysfunction and can influence important clinical aspects of the disease, like motor function itself, fatigue, depression, sleep alteration and quality of life in its entirety (43). On the other hand, phenotypes associated with CNS involvement can be highly different depending upon the age of disease onset (congenital and childhood form vs. adulthood/late onset forms), this reminiscent, in DM1, of how an altered neurofunctional connectivity can be the mirror of either a neurodevelopmental, or a neurodegenerative disorder.

It is unclear whether CNS dysfunction in DM1 patients is, in its nature, a neurodevelopmental, neurofunctional, and/or neurodegenerative disorder. Longitudinal studies on behavior and cognition in DM1 are few but essential for understating the evolution in time of neuropsychological impairment. A follow up study over 5 years (44) shows a worsening in neuropsychological performances and greater cognitive decline than age matched healthy controls. In the 9-years longitudinal study by Gallais et al. (45), the progression in cognitive scores correlated with age and disease duration, but not with nCTG and the rate of decline, which was higher among the late-onset phenotype than in the adult phenotype (earlier onset and longer duration of the disease were associated with greater cognitive deficits). As already said above, it is worthy to remind that neuropsychological functions can decline in a different way along lifespan, as from the study from Gallais et al. (45) which indicates a more precocious progression rate for language and visual memory executive functions compared to visual attention and speed processing, somehow again supporting that CNS involvement in the adult form likely is due to a neurodegenerative process, still not clear whether or not the cognitive decline reported in DM1 is associated with the development of a tauopathy. The expression and the topographic distribution of neurofibrillary tangles in DM1 are similar to what is reported for moderate Alzheimer disease and are higher than that in unaffected individuals of the same age (17).

On the other hand, CNS involvement in congenital and childhood DM1 is due to neurodevelopmental alteration in early life, as longitudinal study showed no further significant decline in cognitive abilities and adaptive behavior over these ages (46).

Brain involvement in congenital DM1 is associated with more severe dysfunctions and is characterized by learning disabilities, intellectual disability, delayed development and psychiatric complaint like phobia, mood disorder and anxiety (47, 48). Attention deficit hyperactivity disorder (ADHD) and autism spectrum disorders were also reported in congenital DM1 (49). CNS alterations influence some aspects of motor skills, besides muscle weakness and myotonia. Recently, Naro et al., have showed that gait impairment in patients with DM1 depends also on a muscle network deterioration, secondary to signal synergy deterioration; they suggested that, beyond weakness and myotonia, muscle connectivity deterioration has a pathophysiological role in gait and postural abnormalities, due to peculiar patterns of fronto-parietal and cerebellum-cerebrum disconnection and disorganized projections from premotor to motorcortical network (50).

As an example of how CNS involvement can influence some canonical primary muscular complaints is data indicating that structural and functional brain changes may also influence fatigue in DM1. A previous 3- T MRI (T_1_/T_2_/diffusion-weighted) study found a correlation between brain hypoechogenicity of the raphe nucleus with fatigue in DM1 and an inverse correlation between fatigue and the extent of brain white matter hyperintensities (51, 52). In neurophysiological studies, both motor cortex excitability, valued by means of transcranial magnetic stimulation measuring intracortical facilitation and inhibition of motor evoked potentials, and central motor conduction time, are also affected in patients with DM1 (53, 54).

Increasing evidence from the literature indicate that brain structural and functional abnormalities account with a dominant role for at least some of the specific neuropsychological dysfunctions in DM1 patients. At brain MRI, Diffusion Tensor Imaging (DTI) and Voxel Based Morphometry (VBM) techniques show corticospinal tract involvement, as shown by Park et al. (55) who described a significant correlation between gray matter volume loss in the sensorimotor cortex and white matter micro integrity alteration of corticospinal tract (valued with DTI parameters) and motor parameters such as muscle strength (valued by Medical Research Council scale), 6 min walking test (6MWT) and handgrip, suggesting a role of corticospinal alteration in motor performance in DM1. Also, deep gray matter alterations (caudate, pallidum, hippocampus, subthalamic nucleus, thalamus, and substantia nigra) has been described, with significant relationship with motor function, sleepiness and cognitive functions, particularly attention and executive function (56). CNS involvement also plays a role in sleep disturbance, with prominent REM sleep dysregulation and a narcoleptic-like phenotype in DM1 (57).

### Neuroimaging in DM1

Steinert's disease is the most frequent form of muscular dystrophy in adulthood and it is characterized by a multisystem involvement. Over the years, several neuroimaging studies aimed at assessing the involvement of the CNS in DM1 patients have demonstrated the presence of anomalies affecting both the gray and white matter. The first conventional magnetic resonance imaging (MRI) studies have shown diffuse brain atrophy in DM1 patients compared to healthy controls, and hyperintense lesions affecting the white matter (39, 58, 59). Subsequent studies have focused on more in-depth analysis through the techniques of Voxel based morphometry (VBM) and diffusion tensor imaging (DTI). The VBM methods have in fact allowed to define a quantitative analysis of the loss of the white and gray matter and to identify the areas in which the loss of gray matter was higher, pointing out a prevalent involvement of the frontal, parietal lobes, middle and upper temporal gyrus and, at the subcortical level, of the basal ganglia and thalamus (51, 59, 60).

Hyperintensities of the white matter have been described in most of the patients with DM1, and typically are bilateral, asymmetric, and mainly affecting the frontal, temporal and parietal lobes, and some authors have found them in the occipital lobes and anterior temporal poles. An enlargement of the Virchow-Robin spaces was also noted (51, 61, 62).

Instead, DTI methods analyzing the microstructure of the white substance, showed widespread reduction of fractional anisotropy (FA) and increase in mean diffusivity. Fractional anisotropy reduction involves all four lobes and particularly uncinate fasciculus, forceps, cingulum inferior and superior longitudinal fasciculus. Using tract based spatial statistics, decreased FA affected both association, projection and commissural fibers. Higher Muscular Impairment Rating Scale (MIRS) was associated with lower FA, and patients harboring higher CTG triplets showed a decrease of FA in the fibers, starting in bilateral prefrontal areas, anterior cingulate and temporal cortex insula and putamen (61, 63). Ates et al. in 2019 with three Tesla MRI studied iron accumulation in deep gray matter nuclei and they found widespread iron accumulation in caudate, pallidum, hippocampus, subthalamic nucleus, thalamus, and substantia nigra; interestingly these alterations were significantly associated with DM1 common symptoms as muscular weakness, daytime sleepiness, and specific cognitive deficit (56).

Brain CT studies also confirmed generalized brain atrophy, and showed frontal hyperostosis and ventricular dilation (61, 64, 65). Ventricular dilation and macrocephaly has also been described by ultrasound studies in newborns in congenital forms of DM1 (61).

Positron emission tomography (PET) showed global glucose hypometabolism, particularly affecting the frontal and temporal lobes; SPECT study confirmed global cerebral hypoperfusion with frontotemporal predominance (60, 66).

Some studies investigated the relationship between genetic parameters and brain alteration, with a positive correlation between longer CTG repeats and greater structural and functional alteration of the brain and muscular impairment (67, 68). MRI studies have focused on the correlations between morphological brain abnormalities and cognitive, neuropsychological and social functions, intelligent quotient (IQ) estimate, attention, visuo-constructive and executive performances, and neuropsychological scores which are associated with both global and regional volume decrease, mainly distributed in the frontal, parietal and subcortical regions (63, 69, 70).

Recently, Gliem et al., in a 5-year longitudinal 3T-brain MRI follow-up study of a group of middle-aged adult-onset DM1 (and DM-2) revealed no significant progression of changes affecting both gray and white matter of the central nervous system over time, hypothesizing a very slow progression or even a stable course of the disease from this point of view in such a patient sample (71).

### Cognition Involvement in DM1

Steinert's disease is characterized by a documented involvement of the central nervous system (CNS) and, also, a neuropsychological functioning impairment so much that over the years, the existence of a “DM1-related-dysexecutive-syndrome” has been proposed (39, 42). Several CNS imaging studies, using different imaging techniques, have demonstrated that DM1 patients have brain abnormalities, in particular: (i) a diffuse brain and gray matter (GM) atrophy affecting the frontal, orbitofrontal, temporal and parietal lobes, anterior insular, post cingulate, brainstem nuclei, basal ganglia, hippocampi, parahippocampal, fusiform and lingual areas, thalami, putamen and corpo callosum (39, 51, 60, 70, 72–77), (ii) a non-specific alteration of white matter (WM) and ventricular enlargement (51, 66, 78), (iii) an hypometabolism and hypoperfusion of frontal and temporal areas (51, 66); (iv) and microstructural damage of WM in every cerebral lobes and in corticospinal and limbic pathways (51, 79); (v), and the enlargement of the hippocampus and amygdala (68).

Although the mechanism underlying CNS involvement in DM1 has not been fully elucidated, some studies suggest that it depends upon a disconnection of the cortical regions, secondary to of the WM tract breakdown (51, 69, 74). To date it is not yet known whether those CNS involvements in DM1 patients are caused by CNS developmental defects, neurodegenerative processes or both.

### Cognitive Domains in Juvanile Form

The juvenile form of Steinert's Disease is characterized by not ever presence of the classic neurological and motor manifestations, but rather manifesting with the invariant occurrence of cognitive deficits or psychiatric disorders which, in most cases, constitute the onset symptoms of the disease which induce parents to consult the specialist. However, only few systematic studies have investigated this aspect of the disease, despite the crucial role played by cognitive deficits and psychiatric disorders on children's lives.

A review conducted by Douniol et al. in 2009 found that the most common symptoms reported in juvenile forms of DM1 patients was learning disabilities. In particular, in two-thirds of DM1 juvenile patients, learning disabilities correlated with intellectual disability, attention deficit, visual spatial impairment and low cognitive speed. DM1 juvenile patients also showed school difficulties in 69–86.7% of cases and academy delay (80–83). The study conducted by Gossend (80) showed that from 21 patients who frequented school, 81% of these were in special education program and only 18% were in normal school programs. Cohen et al. (81) found that DM1 juvenile patients showed impaired written language skills, although there was no intellectual disability. In particular these patients exhibited reading and spelling impairments, even in presence of a normal word identification. These findings don't seem to correlate with a phonological deficit but with the impaired facial expression and oral motor dysfunction, also with the motor coordination disorder and/or a visual-spatial deficit. Recently Caso et al. (74) found an highest impairment in executive functions in the juvenile forms of DM1 patients respect the adult form, regarding especially phonemic fluency. Another study conducted by Woo et al. (84) found that juvenile DM1 patients, compared to adult DM1 patients, have a significant decline in every component of memory domain including immediate, delayed recall and recognition but not in the executive functioning.

Overall, the profile of the cognitive evaluation of juvenile DM1 patients seem quite consistent with that found in patients with adult form of DM1, as described in the following paragraph.

### Cognitive Domains in Adult Form

Despite DM1 being defined as a muscular disease, is well known that neuropsychological functioning is a crucial feature of this disease (39). Neuropsychological functioning is variable from the subtypes of DM1, indeed congenital forms shows higher impairment compared to adult-onset DM1 patients (48). However, patients with adult-onset DM1 very frequently experience cognitive deficit that can be mostly correlated with frontal pole functioning (45, 85, 86).

Additionally, several previous studies have found a significantly selective impairment of neuropsychological functioning in DM1 patients respect of healthy controls, in particular regarding executive functioning, attention, visuo-spatial and visuo-constructive abilities and perceptual reasoning (39, 74, 87–89), with an apparent saving of verbal skills (39, 42, 69, 90). Some studies claim that in DM1 patients with relatively small CTG repeat expansions, cognitive decline may be the only clinical manifestation observed in these patients (85). A study conducted in 66 DM1 patients divided in 22 juveniles and 44 adults showed that there is an impairment in visuo-spatial, visuo-constructive, executive abilities and in naming and visual memory abilities in more than half of DM1 patients (88). Another study showed that DM1 patients have a heterogeneous cognitive profile independent from gender consisting in a significant decline in executive and amnesic domains with a particular visuo-spatial implication and an apparent saving of verbal abilities (69). In particular, this study found that the executive functioning patients showed more deficit in using attention in order to inhibit automatic behavioral responses, by using environmental feedback to shift cognitive sets in using behavior to achieve a goal and in modulating the persevering response. These finding seem suggestive of dissociation from verbal to spatial abilities (69). Also, a study conducted by Cabada et al. (91) found the major deficit in visual-constructive, visuo-spatial functions, and alternating attention in 42 DM1 patients compared to 42 healthy controls. A 9-year longitudinal study that analyzed cognitive functioning in adults with DM1 showed stable verbal fluencies and intelligence quotient and a significant decline over the study period in processing speed in complex executive functioning tasks, verbal memory, and visual attention. Those finding are major in the overall group of adult and late onset DM1 subjects respect to the adult onset group and the percentage of all patients with those cognitive decline increased over the study period (45). A recent review conducted by Okkersen et al. showed that DM1 patients exhibited a significantly worse performance in every neuropsychological domains tested, compared to controls. In particular, it was found that large effect sizes (0.76–1.01) for global cognition, intelligence, visual memory, visuo-spatial perception, visuo-construction, psychomotor speed and social cognition and a small to medium effect sizes (0.33 to 0.66) for language, executive functioning, overall and verbal memory, and attention. It also found that there is a moderate to high statistical heterogeneity in every domain tested except for intelligence, social cognition and global cognition (61). Recently, Fujino et al. evaluated the cognitive functioning of 60 DM1 patients and found that more than half showed several cognitive impairment in particular regarding executive functioning, processing speed, visuo-constructive abilities, attention and working memory (92).

### Intelligence

It is known from previous studies, that DM1 patients have a lower intelligence and an intellectual disability compared to normal control subject in all congenital, childhood and adult onset forms of the disease. The presence of an intellectual disability has been reported by Bird et al. (93) who showed a significantly lower intelligence quotients (IQs), both in adult and congenital onset DM1 patients. Other more recent studies (48, 49) found that patients, children and childhood, with mild-severe DM1 congenital form show an intellectual disability. In particular a learning disability was found in 95% of the severe congenital group, 83% of the mild congenital group, and 89% of the childhood DM1 group, globally the severity of DM1 form correlating with a lower IQ total score (49).

Regarding the global, verbal and performance intelligences it was found that intellectual impairment, consisting in low IQ scores both in least severe forms and was also among the classic adult onset forms of DM1 patients, compared to the normative sample mean. In particular this study found that the total mean of global intelligence was 82.6 corresponding to a low IQ average that decreased with disease duration and the increase of the expansion of an unstable trinucleotide cytosine-thymine-guanine (CTG). Mild DM1 patients showed higher scores in global, verbal and performance intelligences than classic adult onset forms. This study also indicated that low IQ scores do not correlate with motor impairment (90).

Recently, Woo et al. (84), in comparing juvenile with adult DM1 patients, found no differences between the two groups in the IQ, while the verbal test was statistically different, juvenile DM1 patients showed lower scores than adult ones, in any case appearing to show a sort of continuum in CNS clinical manifestations along the natural course of this complex multisystemic muscular disorder.

### Correlation Between CNS Involvement and Cognitive Deficit

Although both CNS involvement and impaired neuropsychological functioning have been proven, only a few studies have investigated the correlation between those relationships with discordant results (51, 60, 66, 74, 79). The assessment of the functional consequences of the CNS involvement is crucial to evaluate the clinical impact (94).

In order to evaluate the correlation between hypometabolism and functional alteration of prefrontal cortex (PFC) in DM1 patients, Caliandro et al. (95) used a functional near-infrared spectroscopy (fNIRS) during the assessment of a phonemic verbal fluency task (pVFT), and found that DM1 patients showed a lower bilateral activation of the PFC, not associated with cortical atrophy, during the task compared to controls that seems to corroborate the hypometabolism of PFC hypothesis. A study conducted by Baldanzi et al. (42) founded: a correlation between brain parenchymal fraction and visuo-spatial and executive performance; a negative relationship between delayed verbal memory and the grade of atrophy in left postcentral, left middle and inferior temporal gyri and left supramarginal gyrus; a negative relationship between radial diffusivity WM alteration and delayed verbal memory, visuo-spatial memory and spatial organization and visuo-constructional skills; and a significant negative relationship between axial diffusivity and immediate and delayed verbal memory. In addition, a study conducted by Gourdon and Meola (96) showed that the impairment of executive functions, episodic memory and spatial and visuo-constructive abilities was correlated with a widespread atrophy in brain areas. Cabada et al. (91) found a significant correlation between visuo-spatial deficit and a WM major rate lesion, ventricle enlargement and volume loss in the central and anteromedial corpus callosum, bilateral cingulated isthmus, right lateral occipital and right pericalcarine cortex in DM1 patients. A recent review conducted by Minnerop et al. (94) showed that many correlations between brain areas and cognitive functions, especially between flexible thinking and GM volume in the secondary visual cortex; visuo-spatial deficit and volume loss in cingulate isthmus, corpus callosum, right occipital, and pericalcarine cortex and ventricle enlargement, and the delayed recall's component of verbal memory and temporal gyri and supramarginal gyrus. A recent study found an inverse association between perceptual reasoning and processing speed with hippocampal volume in DM1 patients compared to healthy control, that may underlie a specific association from cognitive deficits in adult-onset DM1 and the increased hippocampal volume (77).

### Behavioral Impairment in DM1

In the last years, pathologic behavioral patterns are frequently reported in DM1 patients, besides cognitive impairments, such as behavioral disorders, apathy, distinctive personality traits, anosognosia, worsening affective and social cognitive symptoms and daytime somnolence (independently by the presence of sleep disordered breathing) (97), and they have been associated to a poorer quality of life (39, 42, 60), in particular to social and interpersonal difficulties in daily life. Indeed, current studies concerning this topic are consistent and show that these patients have lower social engagement, more psychosocial problems and poorer psychosocial well-being (98–101). Behavior abnormalities were noticed for the first time by Steinert in 1909 and successively several descriptions of suspicious attitude, egocentricity, disagreeableness or indifference (67, 80, 102, 103) or mood disorders (104) were published. Moreover, studies have shown that, as well as for cognitive deficits, behavioral profiles depend on the age of onset. Congenital form is often associated with developmental behavioral disorders, such as autism spectrum disorder, attention deficit and hyperactivity disorder and anxiety disorders (49, 80, 105). Learning disorders and peer problems are present in infantile and juvenile onset, but they are typically milder than those seen in congenital onset (106). Moreover, with DM1 is also reported alexitimia (48).

While, social cognition deficits are detected in adult onset form, and mainly characterized by dysexecutive behavior, involving harm avoidance (like reluctance to seek new experiences, make new friends or form intimate relationship), a lack of cooperation and empathy, inflexibility and apathy (40, 67) and characteristic personality traits (107).

This chapter focuses on adult onset form of DM1, so most of the cited studies have been conducted on these patients. We summarize findings about personality disorders, depression and apathy, social cognition impairment and anosognosia, and their effects on quality of life. Finally, we reflect on importance of psychological well-being in the clinical management of these patients.

### Personality Traits

Clinicians have noticed that DM1 patients have or a tendency to be either obsessive in their health-related care, continuously consulting their referring physician, or contrary avoidant and passive in their attitudes toward health care (39). Currently, a high prevalence of dysfunctional personality has been described and reported in 20–64% of DM1 patients (40, 41). Although previous studies are congruent about the increased frequency of personality disorders in DM1, there are discrepancies on the type of personality traits involved in this disease. In the last 30 years, there were few systematic studies on personality function in DM1 with heterogeneous results. Instead, the most part of research on this topic have been obtained from different data reporting avoidant (40, 67, 103), dependent (108, 109), depressive (93, 108), hypochondriac (93, 110), obsessive-compulsive (103, 111), passive- aggressive (103), aggressive-sadistic (107), but also paranoid (103, 107, 109, 110), schizotypal (103), and schizoid (93) personality traits.

We analyze the most recent studies between these ones, in Meola et al. (40) report, their results show that although none of the tested patients has fulfilled the criteria for the diagnosis of avoidant personality disorder, DM-1 have displayed significant avoidant behavioral traits compared to control subjects. In general, almost all patients were reluctant to make new friends, carry out new activities, or take personal risks, and the majority of patients were employees with no responsibility or decision-making positions. Results of Winblad et al. (67) are in line with previous data. Authors have indicated the presence of deviant personality in 20% of DM1 subjects, and in particular higher scores on Harm avoidance and lower on Persistence, Self-directedness and Cooperativeness in comparison with healthy controls (HC) and to patients with other neuromuscular disorders, highlighting tendency to prefer loneliness and not function well in social groups. Different conclusions are reported in the largest study which included 121 patients, it evidences aggressive/sadistic (rash, dogmatic, hostile, competitive, pernicious and explosive behavior) and paranoid traits (thoughts rigidity) (107). Partially different results are reported in Peric et al., work's (109) evaluating 62 patients. They have described the presence of at least one pathological personality trait in 75.8% of patients, and in 58.1% after clinical interviews. The most common personality trait was dependent (51.6%), followed by paranoid (38.7%). (110) have partially replicated previous results in 27 patients, whereas 89% of patients reported elevation on at least 1 clinical scale, and specifically, the most common elevated clinical scores across all patients include paranoia, like previous results, but also schizophrenia and hypochondriasis. Despite its name, schizophrenia scale does not usually indicate the presence of this disorder, but it describes the presence of mental and emotional confusion. Recently, Paunic et al. (111) have published the results of their study, where 44 DM1 patients are a control group compared to 27 genetically confirmed DM2 patients. Their results confirm indirectly, the presence of typical personality traits in DM1 because in DM2 group there was not only a scale with pathological scores, but also, DM2 patients had lower scores compared to DM1 patients in almost all scales.

Concerning the interpretation of personality abnormality there are two hypotheses: the first one is that personality traits could be a direct consequence of DM1 pathology affecting the brain, as shown by atypical connectivity in the default mode network associated with schizotypal-paranoid traits (110) or a severe involvement of the dorsolateral prefrontal cortex, cingulum, medial and lateral parietal regions, occipital and temporal lobes, that has been associated with personality changes in many neurological and psychiatric diseases (74). Second hypothesis is that psychological symptoms arise as a consequence of DM1 clinical manifestations, because factors such as pain and fatigue could also bring patients to avoid social situations and eventually to develop avoidant or schizotypal personality traits in response to the disease.

Above all, there are two strength points: the first is that the prevalence of personality disorders in DM1 is higher than in the general population (that are 6%) (112). The second is that personality traits, and in particular paranoid personality traits have a negative effect on the quality of life (109).

### Social Cognition Impairment

Social cognition deficit seems to be particularly present in childhood onset, given that in this one it has often been detected as autism spectrum disorder (49); while few studies have tried to investigate social cognitive function in adult onset (113–115), they often have severe difficulties in daily-living activities including social interaction. Theory of mind (TOM) consist in having a good relationship with others in social context and is probably caused of social interaction deficit in DM1. For this reason, the interest on this topic has increased in the last years and different studies have reported TOM deficit (113, 114, 116, 117). TOM is composed by a cognitive and affective part, with different functional substrates. One study (116) has demonstrated that DM1 patients are more compromised in the affective aspects of TOM than in cognitive parts, because both have the ability to infer the mental states of others by looking at their eyes and the capacity to understand when remarks are inappropriate, are impaired. Both of them have an emotional impact, but on the contrary, patients are able to take other's perspective and understand their interactions or beliefs, which corresponds to the cognitive aspect of TOM. Instead, another study has proved otherwise (117), finding pathological scores in both test assessing TOM in 80% of sample, and, a more remarkable impairment at TOM story than in Reading the Mind Eyes Test (RMET). Thus, it suggests a weakening of cognitive TOM and characterize the highest executive functions underlying this one (118). Other studies have assessed the integrity of social cognition with facial emotion recognition tasks (113, 114, 116, 119, 120) (Kobayakawa et al., 2016). A lot of studies, analyze the ability to recognize each emotion separately and have revealed the same results: DM1 patients scored significantly lower than HC on the recognition of negative emotion, such as anger, disgust (119) and fear (113). Interestingly, one study has found that the presence of difficulties in processing happiness (120). Moreover, it has been discovered (119) that the facial emotion recognition scores correlate negatively with age, supporting the notion, recently emerged that in DM1 there are age related decline of frontal and temporal functions (44, 45, 85, 121). It is known that facial emotion recognition impairment is a core and early developing feature in fronto-temporal dementia (122, 123), so, an important question is still emerging: is emotion recognition a deficit marker of a decline in fronto-temporal functions in DM1 patients? The role of this deficit as a marker of aging related decline is discussed.

Some of these studies (110, 114, 115, 117, 124) support the hypothesis that social cognition impairments are a direct consequence of brain abnormalities and not a reaction symptom. Specially because it has been found that subnormal facial and emotional recognition correlates with the size of CTG expansion. So, social, emotion and recognition impairment seems to be a sensitive domain in DM1 (125). For example, in previous studies (115), an association has been found between lesions in the frontal, temporal, and insular sub cortices and decreased emotional sensitivity to disgust and anger among DM1 patients. Another work has highlighted the increase in connectivity in the left fusiform gyrus (FG) that might be associated with impairment in face perception and TOM (110). The same authors, 2 years later (117) discovered an association of TOM scores with specific patterns of abnormal connectivity between the left inferior temporal and fronto-cerebellar nodes in DM1 brains, suggesting that social cognitive impairment in DM1 patients is attributable to impaired emotional processing linked to white matter lesions. In particular, the frontal insula Von Economo neurons seem to be involved in social and emotional behavior (126).

A recent resting-state functional MRI 3T study investigated ventral tegmental area connectivity during subject assessment with the IOWA Gambling task, finding a prominent decision-making deficit in patients with DM1. Moreover, this deficit could be related to increased connectivity between brain areas critically involved in the reward/punishment system and social cognition and ventral tegmental area, one of the major sources of diffuse dopaminergic projections in the brain (127).

All these data are relevant not only for a better pathophysiological comprehension of DM1, but also for non-pharmacological interventions to improve clinical aspects and impact on patients' success in life.

### Awareness Impairment

Presence of neuropsychological and behavioral difficulties can bring to reduce awareness of disease burden and progression, and certainly low compliance to treatment. Baldanzi et al. (69), comparing DM1 patient Individualized Neuromuscular Quality of Life Questionnaire (INQoL) self-ratings with the main caregiver's reports, has highlighted that an elevated percentage (51.6%) of DM1 subjects was disease unaware. In particular, patients tended to understate some aspects of their psychosocial difficulties, especially Independence (52.4%) and Social Relationship (47.5%), suggesting an impairment in self appraisal of their adaptive behaviors and interaction with the environment. The importance of these results is about achievement of an awareness characterization at single domain specificity level. Moreover, lack of awareness results significantly related to the performance failure in cognitive test, in particular cognitive flexibility and visual-spatial memory, in line with some theoretical models that identify self-awareness as a metacognitive function relying on high-order cognitive abilities localized in frontal circuitry and parietal structures (128, 129).

### Psychological Functioning: Apathy, Depression, and Anxiety

A recent meta-analysis (130) has estimated pooled prevalence of clinically significant levels of symptoms of depression (19%), anxiety (17%), and apathy (55%) in DM1. Concerning anxiety, different types of anxiety disorder were observed: generalized or separation anxiety disorder and specific phobias (105). Although, some authors have reported that frequency of mood disorders in DM1 is not higher than in general population or in other neuromuscular disease that does not affect the brain directly (41, 131), few studies have shown that significant depressiveness in DM1 patients was more common than in HC (51, 104) and in other neuromuscular disorders (132). However, even when symptoms of depression and anxiety are registered, only few of DM1 patients meet the criteria for major depression (131) or any psychiatric disorder (40), probably because it is a more pronounced somatic than cognitive-emotional dimension of depression (41). Moreover, it is unclear whether depression is primary or secondary. It has been detected (74) that an association between WM brainstem atrophy at the level of the basal pons and the middle cerebellar peduncles, which can thus be involved in depression and emotional control (133), corroborant the hypothesis that depression in DM1 has at least in part, a pathomorphological correlates and demonstrates that it is not only a reactive phenomenon (74). Another common neuropsychiatric feature detective in DM1 are lack of interesting or also named apathetic behavior (99, 134). One study (99) has compared the level of apathy in DM1, Facio-Scapulo-Humeral-Dystrophy (FSHD) patients and in controls, demonstrating that the global score of apathy was significantly higher in DM1 patients than comparison groups. Indeed, 39.5% of DM1 patients met the criterion for apathy, contrasting with only 21.1% of FSHD patients; while, no control subject was apathetic. Results also show that apathy in DM1 is independent of the psychopathological domain, but associated to general cognitive status, suggesting a central cause for apathy in DM1 rather than an adjustment process to cope with the progressive and debilitating nature of the disease. Thus, a better comprehension of apathy and its outcomes in DM1 is relevant, and, in this regard, a clinical routine evaluation is advised.

## Methods to Assess CNS Involvement in DM1 as Possible Outcome Measure

In clinical practice, as well in research, an outcome measure is a sized mean used to evaluate the effect, both positive or negative, of an intervention or treatment. Outcome measures objectively determine the function of a patient when at the beginning of a clinical trial, and significantly quantify the deviation progress and efficacy of a therapeutic intervention, not only in terms of pure numbers but rather as parameters able to perform the real burden of the disease. Outcomes measures can be then obtained from clinical examinations, laboratory or imaging or functional test or patient-reported.

However, despite the number of studies published in the literature on this topic, there are not comprehensive enough consensus protocols for neuroimaging and cognitive testing in DM1. Recent international workshops stressed the needs to design patient questionnaires and clinical neuropsychological tests to better size and confidently reproduce what cognitive domains of CNS are affected in DM; moreover, more longitudinal natural history studies with CNS measures are strongly needed to understand the pattern of progression and decline in DM1 over time (135). Finally, we also need terms of comparison along longitudinal time-elapsed studies between neuroimaging and neuropsychological data that will help to correlate any decline in cognitive functioning with a change in brain morphology. A consensus for that has been reached on some biomarkers for cerebral involvement in DM1, as from recent workshops that also have risen the needs to create an international DM registry focused on CNS aspects and to collect, for this purpose, blood, muscular tissue, fibroblasts and cerebrospinal fluid in dedicated biobanks (125, 136, 137). These issues need to be resolved in order to increase readiness for clinical trials in DM.

Following all the above considerations, it comes out that, although with a certain degree of variability within the complex and heterogeneous clinical picture that characterizes DM1, CNS involvement appears nonetheless holding a relevant role in depicting the severity degree of the disease in its entirety as well as its natural course history, reproducing with acceptable assumption illness trajectory. This in turn rises the important question as to whether incorporating clinical or instrumental indices related to CNS involvement into reference outcome measures for treatment interventions can be a valuable tool. Indirect data suggest that this can be the case, as for instance when considering the different scales of health-related quality of life (HRQoL), included those patient-reported, for scores involving most of the considered domains, muscle weakness, pain, fatigue, activities, independence, social relationships, emotions and body image. Nonetheless the general lack of psychometric data still limits the significance of these scales to faithfully figure out the stage of the disease (43).

### Indices of Cognitive Impairment

As it is well known, there are many methods for evaluating the neuropsychological functioning of DM1 patients. A recent review conducted by (78) indicated the instruments most used in the studies present in the literature, listing them according to the frequency with which they had been used.

**Assessment of verbal-auditory short and long-term memory:** Immediate and Delayed Recall (IR, DR) of Rey Auditory Verbal Learning Test (RAVLT) and Digit Span Test forward and backward.

**Assessment of visual-spatial short and long term memory:** Copy and Recall (C, R) of Rey Osterrieth Complex Figure (ROCF), and Corsi Block-tapping Test (CBT).

**Assessment of selective and shift attention and automatic response inhibition:** Trail Making Tests (TMT-A and TMT-B) and Stroop Test.

**Assessment of frontal and executive functions:** Phonemic verbal fluency test (FAS), Frontal Assessment Battery (FAB) and Wisconsin Card Sorting Test (WCST).

**Assessment of visuo-constructive abilities:** Rey-Osterrieth Complex Figure copy (ROCF-C); Object assembly and Block Design WAIS subtests.

**Assessment of psychomotor speed:** Wechsler digit symbol coding (WDS) and Trail making test, part A (TMT-A).

**Assessment of intelligence:** Raven progressive matrices (RPM) and Wechsler adult intelligence scale (WAIS).

### Behavioral Impairment

Behavioral impairments have been assessed with neuropsychological and psychological instruments. In regards to personality disorders, it is possible to use personality inventory such as Minnesota Multiphasic Personality Inventory (MMPI), administered in some studies (93, 110, 138) or Millon Multiaxal Clinical Inventory (MMCI), also used in other (107, 109, 111). Both of them are based on Diagnostic Criteria of DSM. Another instrument based on DSM criteria is the Structured Clinical Interview-Personality Disorders (SCID-PD), actually at fifth Version. This instrument has been used in Meola et al. (40) and in (139) studies. In other works (67, 140) questionnaires have been used based on biopsychosocial model of personality such as Temperament and Character Inventory (TCI) or inventory based on Big Five model like as questionnaire like NEO five-factor inventory (NEO-FFI).

To assess social cognition, the majority of the studies (116, 117) have used Reading the mind Eyes task (RMT), Faux Pas Test [in (116) and in (119)] also TOM story task [in (117)]. Serra et al. (120) have used a Social Cognition Battery, while in other researches a test has been used to assess Facial Emotion Recognition ability such as the Facial Emotion Recognition Test (POFA), or report questionnaire measure cognitive and affective empathy (TECA) [in (119)].

Individualized Neuromuscular Quality of Life questionnaire (INQoL) (141) has been often used as a measure of QoL [in (69, 88) and in (109)]. To evaluate the level of depression and anxiety, they have been used principally two questionnaires, like Hamilton scales for depression and anxiety (HamD and HamA) [in (88)] and Beck Depression Inventory (BDI) [in (41) and in (74)]. Finally, to measure the presence of apathy the Apathy Evaluation Scale [in (99)] has been used to asses incidence of anosoagnosia (69), and a comparison has been done to evaluate the statistical agreement either between the severity of motor impairment scored by MIRS (administered by a trained clinician) and symptoms complained by patients and assessed by INQoL Weakness Domain (administered by a psychologist) or between Insole separately administered to both patient and to the main caregiver.

As technology is becoming an increasingly more important part in the medical field, new tools and instruments are available for health professionals to assess in a more real life complex behavioral-cognitive profiles. One of these new technologies is represented by serious games, games developed with a primary purpose other than entertainment and are currently being used in several fields, from education, social and environmental contexts, to health care areas. Firstly applied in mild cognitive impairment, but also in normal aging, they have recently found space in the purpose of assessment, follow up and rehabilitation for different pathologies, in addition to conventional treatment and rehabilitation (142). Many other potential applications are currently being studied for these tools, for instance, to assess executive functions (143), a reason why new technology appears potentially suitable for DM1, eventually in addition with physical stimulation, indicating (unpublished data) a promising usefulness as quantifiable and comfortable method of clinical evaluation in a domestic environment.

### Neuroimaging Outcome Measures

An international consensus on neuroimaging outcome measures in DM1 does not exist, however, the published case studies and literature reviews have described numerous resonance parameters to be evaluated, the evolution of which over time is however uncertain. The most frequently applied techniques are morphological MRIs, evaluating brain atrophy and white matter affection, with a few studies using functional MRI (136). Additional longitudinal neuroimaging studies will permit determining the natural history of the CNS affection and to help to differentiate between developmental defects, neurodegeneration, and age-related lesions (136). A standardization of software and of MRI analysis method is strongly needed.

According to the 2014 workshop report of the DM-CNS group, among the most used MRI techniques, the quantification of brain volume by voxel-based morphometry (VBM) automated segmentation procedure of T1-weighted MR images has a dominant role and allows an objective calculation of gray and white matter volumes and abnormalities (125).

Regarding quantification of global and regional brain volume, numerous parameters are described such as the brain-parenchima factor (BPF, the ratio of brain parenchymal volume to intracranial volume, Kassubek), callosal volume (144), the gray matter to white matter ratio (145). As regard to quantification of white matter abnormalities, the most widely used techniques are signal changes evaluation on T2-weighted images (62), diffusion tensor imaging (DTI) study parameters (fractional anisotropy, mean diffusivity, radial and axial diffusivity) (79). Other possible imaging outcome measures are proton MR spectroscopy evaluating single-voxel brain metabolities [N-acetylaspartate, phosphocreatine, choline and lactate, (146)], glucose consumption on 18FDG PET, cerebral blood flow on SPECT (40), Table 1 summarizes clinical instruments in the considered domains to assess CNS involvement in DM1.

**Table 1:** Central Nervous System involvement in DM1: tools for cognitive and behavioral measures and for Neurological/neuroimaging assessment.

**Cognitive section**	
Verbal-auditory short and long-term memory	Immediate and Delayed Recall (IR, DR) of Rey Auditory Verbal Learning Test (RAVLT) and Digit Span Test forward and backward
Visual-spatial short and long-term memory	Copy and Recall (C, R) of Rey Osterrieth Complex Figure (ROCF), and Corsi Block-tapping Test (CBT)
Selective and shift attention and automatic response inhibition	Trail Making Tests (TMT-A and TMT-B) and Stroop Test
Frontal and executive functions	Phonemic verbal fluency test (FAS); Frontal Assessment Battery (FAB) and Wisconsin Card Sorting Test (WCST)
Assessment of visuo-constructive abilities	Rey-Osterrieth Complex Figure copy (ROCF-C); Object assembly and Block Design WAIS subtests
Psychomotor speed	Rey-Osterrieth Complex Figure copy (ROCF-C); Object assembly and Block Design WAIS subtests
Intelligence	Raven progressive matrices (RPM) and Wechsler adult intelligence scale (WAIS)
**Behavioral section**	
**Domain**	**Tools**
Personality	Minnesota Multiphasic Personality Inventory (MMPI) Millon Multiaxal Clinical Inventory (MMCI) Structured Clinical Interview-Personality Disorders (SCID) Temperament and Character Inventory (TCI) Big Five model like as questionnaire like NEO five-factor inventory
Social Cognition	Reading the mind Eyes task (RME) Faux Pas Test TOM story task Social Cognition Battery Facial Emotion Recognition Test Report questionnaire measure cognitive and affective empathy (TECA)
Quality of Life	Individualized Neuromuscular Quality of Life questionnaire (INQoL) Health-related quality of life (HRQoL),
Depression and Anxiety	Hamilton scales for depression and anxiety (HamD and HamA) Beck Depression Inventory (BDI)
Apathy	Apathy Evaluation Scale
Anosoagnosia	Patient/observer comparison of clinical severity impairment and patient/caregiver comparison of subjective clinical symptoms
**Neurological section**	
Brain morphology	Magnetic Resonance imaging (MRi) Voxel Based Morphometry (VBM) Diffusion Tensor Imaging (DTI) Magnetic Resonance spectroscopy (MRS)
Multidimensional self- reported scale	DM1-ActiveC Myotonic Dystrophy Health Index

### CNS Outcome Measures: Why a Myologist Can Still Remain a Neurologist in Caring of CNS Involvement for Clinical Trials in DM-1

In 2011 and subsequently in 2013, the first workshops on outcome measures in patients with type 1 myotonic dystrophy were carried out with the aim of outlining outcome measures that can be used in the therapeutic trails of these patients (147, 148). After that, increasing interest has been focused on the attempt to get informative and reliable items able to depict at the best disease trajectory in DM1 (7). Related questions to be dealt with to this regards include, like many other chronic progressive illnesses and in the lack of decisive therapies, the high phenotypic heterogeneity of the disease, existence of far different clinical forms and the very slow progression rate of it from one side; the reliability of the outcome measure in terms of inter- and intra-observed variability, its fidelity to reproduce clinically relevant complain and its capacity, in front of a minimum deviation over time, to be caught with the maximal significance by a given therapy efficacy in a given patient sample size, from the other side. While already difficult for myopathic core features of DM1, the answers to these questions appear even more arduous when dealing with CNS involvement manifestations due to the complexity of their clinical interpretation and evaluation. In their recent study, Gliem et al. (71) found minimal if any modifications of both neuropsychological and 3T brain MRI parameters of a period of 5 years follow up in 16 mean-aged adult form DM1, as well as in an equal number of DM2, patients, indicating that, although frequently informative compared to normal in cross-sectional studies, alterations in CNS parameters can lose clinical significance over time.

As above stated, the main outcome measures actually used to study central nervous system and cognitive impairment in DM1 patients are represented by neuropsychological tests, in particular for frontal functions, attention, executive performance and language, Frontal Systems Behavior Scale, Cambridge Brain Sciences computerized (online) testing, as well as neuroimaging MRI with Voxel based Morphometry and Diffusion Tensor Imaging sequences. A recent study has also shown how the outcome measures currently used for cognitive and central nervous system involvement can be influenced by the peripheral component of the disease, highlighting the need to carry out longitudinal and large case studies to evaluate the best outcome measures to be adopted in DM1 (89).

In addition to these objective outcome measures, a group of self-reported outcome measure are described. Some have been specifically developed for use in DM1 patients, including DM1-ActivC, a measure of capacity for activity and social participation, and the fatigue and daytime sleepiness scale (FDSS) (149, 150). DM1-ActivC, in a recent trial, has been shown to significantly improve after a 10-months trial of cognitive behavioral therapy, which ameliorated ability to complete activities of daily living and social participation (151). The Myotonic Dystrophy Health Index (MDHI) is self-reported rate of symptoms in a multi-domains scale which includes social performance, fatigue and also cognitive complaints (152).

All the above considerations, also taking into account of the clinical relevance of CNS damage manifestations in DM1, underline the necessity to further develop this area of study in the attempt to improve the level of reliability of available biomarkers in this regard. At the same time, this arises a provocative and even paradoxical question on how the myologist, by now the figure in charge to care of a patient with DM1, needs to remain a neurologist to keep his skills so high to better appreciate this important aspect of Steinert disease.

## Conclusions

Preliminary data are present in a recent trial by Okkersen et al. (151). Results show that cognitive-behavioral therapy increased DM1-Active-c score at 10 months, that is a measure of activity capacity and social participation. Moreover, interestingly secondary outcome measures, like total distance on the 6-min walking test, daily activity levels and objective physical activity are significantly improved, whereas fatigue and daytime sleepiness scale score are decreased. Both beneficial effects on primary and secondary outcomes were sustained at 16 months. On the contrary, depression and apathy evaluations were stable (135). So, currently data, although at the moment there is a lack of strength empirical studies on this regard, encourage to provide DM1 patients and their families with neurocognitive rehabilitation as well as psychological support programs (86).

The first one should be focused on attention, memory and executive function, including goal management training approach, already successfully applied in people with neurological conditions, and problem-solving training, metacognitive or self-instructional training that encouraging patients to plan and reduce impulsivity (153). The second one should be focused on disease awareness and coping strategies. It has been demonstrated that dysexecutive syndrome and behavioral problems significantly impact on QoL and it has been suggested that cognitive rehabilitation may improve patients' QoL (88), In particular in adult onset group where QoL is more impaired (109), likely because late onset of the disease causes greater frustration, while an adjustment to the disabling disease since childhood in DM1 juvenile onset.

In conclusion, summarizing all the available literature, data emphasize the importance to evaluate CNS symptoms, including neuroimaging data and cognitive and behavioral measures, in routine clinical management of DM1 patients by searching for suitable outcome measures for that. Tools developed from observational prolonged trials and ultimately targeted to mirror patient's quality of life will be of great importance for considering that (154). Brain dysfunctions could play a crucial role in emerging of most of the clinical features characterizing this neuromuscular disease. Through animal model it was possible identified molecular outputs that could play a prominent role as CFS biomarkers for human DM1 patients (96). In line with this, the majority of existing studies highlights the implication of CNS involvement also in planning rehabilitation strategies targeted to improve aspects that have an impact on quality of life beyond medical management of these patients. The safe of QoL parallel to medical measures, means improving also support of caregiver system and efficiency of the entire assistive system. In doing that, additional knowledge will also come from collaboration initiatives and consortia between different expert centers across the world, as it has been the case of the outcome measures in myotonic dystrophy (OMMYD) group that has been working to reach consensus statements in some key areas over the last few years (148). Although much else still remains to be done, also from this point of view the main objective remains to build upon fundamental synergistic initiatives, for instance, data sharing and global registries, instruments to better define strategies for accelerating trial readiness in DM1.

## Author Contributions

CS, GSp, EL, and GSi: examining scientific literature, participation to experimental approach, and writing the paper. GSi: also supervising conclusive form. LS: contribution to experimental approach. CA and GR: contribution to experimental approach and advise. All authors contributed to the article and approved the submitted version.

## Conflict of Interest

The authors declare that the research was conducted in the absence of any commercial or financial relationships that could be construed as a potential conflict of interest.
